# Comparative analysis of official analytical methods for tocopheryl esters for food additive quality control

**DOI:** 10.1016/j.fochx.2026.104154

**Published:** 2026-06-30

**Authors:** Geun-Hee Cho, Hyun-Woo Oh, Tae-Woong Song, Young-Jae Heo, Su-Jong Kim, Ji-Hyun Im, Xiaolu Fu, June-Seok Lim, Min-Hye Kim, Hee-Jae Suh, Ok-Hwan Lee, Sun-Il Choi

**Affiliations:** a Department of Food Biotechnology and Environmental Science, Kangwon National University, Chuncheon 24341, Republic of Korea; b Department of Food Science and Biotechnology, Kangwon National University, Chuncheon 24341, Republic of Korea; c Department of Food Science, Research Center for Food and Bio Convergence, Sun Moon University, Asan 31460, Republic of Korea

**Keywords:** Food additives, Tocopheryl esters, Method validation, Measurement uncertainty, Fortifying nutrient

## Abstract

Tocopheryl esters are widely used as nutritional fortifiers and antioxidant additives, making accurate quantification essential for regulatory compliance and quality control. However, official analytical methods differ among jurisdictions, which can cause method-dependent variability. Here, the official procedures for d-α-tocopheryl acetate, dl-α-tocopheryl acetate, and d-α-tocopheryl succinate prescribed in Korea, China, and Japan were reproduced and validated according to ICH Q2(R1). Although all methods met the criteria, sensitivity, recovery, and precision differed by analytical principle (GC–FID, HPLC–PDA, and titration). Recoveries ranged from 86.34 to 114.86% (GC–FID), 94.13–99.75% (HPLC–PDA), and 99.86–100.45% (titration), with intra-day RSD below 2.6% for all methods. Measurement uncertainty, estimated using ISO GUM and Eurachem approaches with a coverage factor k = 2, yielded expanded uncertainties (U) of 4.87–10.39%, revealing method-specific contributions from calibration, sample preparation, and repeatability. The discrepancies were linked to methodological design, underscoring the value of comparative evaluation in food additive analysis.

## 1 Introduction

Tocopheryl esters, including d-α-tocopheryl acetate, dl-α-tocopheryl acetate, and d-α-tocopheryl succinate, are widely used as nutritional fortifiers and antioxidant additives in a broad range of food products (Rupérez et al., 2001; Abidi, 2000). As potent lipid-soluble antioxidants, tocopherol isoforms scavenge reactive oxygen and nitrogen species and protect biological membranes from oxidative damage, underpinning their nutritional and functional significance (Es-Sai et al., 2025). Esterification of tocopherols is commonly employed to enhance chemical stability during food processing and storage, as free tocopherols are more susceptible to oxidative degradation (Brigelius-Flohé & Traber, 1999; Górnaś et al., 2022; Huo et al., 1999). Quantitative kinetic analyses confirm that free tocopherols undergo progressive depletion during lipid oxidation in bulk oils, with depletion patterns correlating with the loss of radical-scavenging activity (Cantele et al., 2025), reinforcing the rationale for esterification to enhance stability in food applications. Owing to their extensive use and functional importance, accurate and reliable quantification of tocopheryl esters is essential for regulatory compliance, quality control, and the establishment of internationally consistent food additive standards (Konings et al., 2024). In this regard, reliable analytical data for tocopheryl ester additives form the basis of regulatory monitoring, food additive quality control, and the assurance of safety and efficacy in fortified food products (Castanheira et al., 2006). Furthermore, the growing interest in food bioactives and nutraceutical compounds has emphasized the importance of reliable analytical techniques for quantifying functional components in food matrices (Sajid et al., 2025).

Although tocopheryl esters are authorized for use in many regions worldwide, including under international frameworks such as the Codex Alimentarius, the officially designated analytical methods for their quantification vary among regulatory authorities. In particular, while official methods in the European Union and the United States predominantly rely on harmonized HPLC-based procedures for tocopherol derivatives, notable methodological diversity exists among national standards. Korea, China, and Japan adopt distinct analytical principles and procedural frameworks for determining tocopheryl ester content, making them a representative set of regulatory systems for the comparative evaluation of method-dependent variability. This rationale aligns with broader analytical practice in food science, where method selection is guided by sensitivity, reproducibility, and suitability for the target sample matrix (Iqbal et al., 2025). These three jurisdictions were specifically selected because their official monographs collectively encompass all three principal analytical principles applied to tocopheryl ester determination—HPLC–PDA, GC–FID, and titrimetry—thereby allowing direct comparison of detection mode, separation strategy, and calibration approach within a single experimental framework. In Korea (Ministry of Food and Drug Safety, MFDS, 2024), GC–FID is specified for both d-α-tocopheryl acetate and d-α-tocopheryl succinate, whereas dl-α-tocopheryl acetate is determined using a classical redox titration procedure. China employs GC–FID as the official method for all tocopheryl esters under the Guobiao (GB) standards, emphasizing volatility-based separation and flame ionization detection (National Health Commission of the People's Republic of China and State Administration for Market Regulation, 2016). In contrast, Japan (Ministry of Health, Labour and Welfare, MHLW, 2024) relies exclusively on HPLC-based procedures described in the Japanese Pharmacopoeia and the Japanese Standards for Food Additives. These methodological differences extend beyond detection principles to include extraction conditions, sample preparation steps, chromatographic parameters, and calibration strategies (Huang et al., 2025).

Such heterogeneity in official analytical procedures implies that identical tocopheryl ester products may yield different quantitative results depending on the national method applied, even when analyzed under controlled laboratory conditions. Method-dependent variability may arise from differences in analyte volatility and thermal behavior during GC analysis, matrix effects or extraction efficiency in HPLC-based procedures, or the inherently lower selectivity and operator dependence of titrimetric assays. Despite these plausible sources of variability, few studies have systematically reproduced and compared national official analytical methods using a unified experimental framework, and experimental evidence explaining how procedural differences translate into analytical divergence remains limited. This gap is particularly significant in the context of ongoing international efforts to improve the harmonization and comparability of food additive testing.

In parallel, international quality assurance frameworks increasingly emphasize measurement uncertainty as a quantitative indicator of analytical reliability. Under ISO/IEC 17025 and Eurachem guidelines, measurement uncertainty is regarded as essential for interpreting analytical results, assessing method robustness, and ensuring comparability among laboratories. While measurement uncertainty has been applied in analytical method validation in selected research fields, it has rarely been used to interpret or compare official analytical methods for food additives. Consequently, the extent to which national methodological differences contribute to analytical variability remains insufficiently characterized (Katsa et al., 2021).

To address these issues, the present study systematically reproduces and evaluates the official analytical methods for d-α-tocopheryl acetate, dl-α-tocopheryl acetate, and d-α-tocopheryl succinate prescribed in Korea, China, and Japan. Each method was implemented without modification and validated according to ICH Q2(R1) guidelines. Measurement uncertainty was further estimated using ISO GUM and Eurachem approaches, not as a primary objective, but as an interpretative framework to elucidate method-dependent variability. By integrating method reproduction, validation, and uncertainty-based interpretation, this study provides experimental evidence clarifying structural differences among national official methods and underscores the importance of comparative evaluation for improving consistency in food additive analysis. This study aimed to reproduce and comparatively evaluate the official analytical methods used in Korea, China, and Japan for d-α-tocopheryl acetate, dl-α-tocopheryl acetate, and d-α-tocopheryl succinate, to assess method validation parameters and measurement uncertainty, and to examine the implications of the findings for inter-laboratory comparability and regulatory harmonization.

## 2 Materials and methods

### 2.1 Chemicals and reagents

Certified reference standards of d-α-tocopheryl acetate (≥98.0%), dl-α-tocopheryl acetate (≥98.0%), and d-α-tocopheryl succinate (≥97.0%) were obtained from Sigma-Aldrich (St. Louis, MO, USA). Analytical-grade methanol (≥99.9%), trifluoroacetic acid (≥99.0%), ethanol (≥99.9%), ethanolic sulfuric acid solution, and 0.01 N ammonium cerium sulfate solution were purchased from Daejung Chemicals (Siheung, South Korea).

### 2.2 Samples

Food grade of dl-α-tocopheryl acetate (99%) was obtained from DSM Nutritional Products Ltd. (Basel, Switzerland), and d-α-tocopheryl succinate (98.64%) was obtained from Matrix Life Science Pvt. Ltd. (Aurangabad, India).

### 2.3 Analytical procedures for d-α-tocopheryl acetate

The procedures below reproduce the official national methods for d-α-tocopheryl acetate (Korea, China, Japan) without modification, enabling direct comparison of method-dependent performance. For the Korean method, GC–FID analysis was performed in accordance with the MFDS Food Additives Code. The sample was accurately weighed, dissolved in n-hexane, and analyzed using a capillary column under split-injection conditions. The injector and detector temperatures were set at 290 °C and 300 °C, respectively, with nitrogen (N₂) as the carrier gas. The oven was operated isothermally at 260 °C, and a 1 μL aliquot of the sample solution was injected. Quantification was performed using the internal standard method, with hexadecyl hexadecanoate (cetyl palmitate; CAS 540–10-3) prepared in n-hexane at 3 mg/mL as the internal standard, as prescribed by the MFDS Food Additives Code. For the Chinese official method, GC–FID analysis was performed using an HP-1 capillary column. The injector and detector temperatures were set at 290 °C and 300 °C, respectively, and nitrogen (N₂) served as the carrier gas. The oven was operated isothermally at 260 °C, and a 1 μL aliquot of the sample solution was injected in split mode. Quantification was achieved using the internal standard method, employing hexadecyl hexadecanoate (3 mg/mL in n-hexane) as the internal standard, in accordance with the corresponding Chinese national standard (GB). The Japanese method followed the procedures described in the Japanese Standards for Food Additives (JSSFA). Samples were dissolved in methanol, filtered through a 0.45 μm PTFE filter, and analyzed by HPLC using a Phenomenex Hypersil C_18_ column (250 × 4.6 mm, 5 μm) maintained at 35 °C. The mobile phase consisted of methanol and deionized water (98:2, *v*/v), delivered isocratically at 1.0 mL/min. The injection volume was 20 μL, and PDA detection was performed at 284 nm, with a total run time of 20 min.

### 2.4 Analytical procedures for dl-α-tocopheryl acetate

The following methods for dl-α-tocopheryl acetate follow the respective official monographs; the differing analytical principles facilitate assessment of inter-method variability. The Korean method, based on titration, was reproduced as specified in the Food Additives Code. Approximately 0.25 g of the sample was weighed into a 100 mL brown round-bottom flask, dissolved in 25 mL of anhydrous ethanol, and mixed with 20 mL of ethanolic sulfuric acid solution (3 → 20). The mixture was refluxed for 3 h, cooled, transferred to a 200 mL brown volumetric flask, and diluted to volume with anhydrous ethanol. A 50 mL aliquot of this test solution was then combined with 50 mL of ethanolic sulfuric acid solution (3 → 200) and 20 mL of water. Titration was performed using 0.01 N ammonium cerium(IV) sulfate solution, where 1 mL of titrant corresponds to 2.364 mg of dl-α-tocopheryl acetate. For the Chinese method, GC–FID analysis was conducted using an HP-1 capillary column. The injector temperature was 290 °C with a split ratio of 1:20, and nitrogen (N₂) was used as the carrier gas. The oven was operated isothermally at 290 °C, and the detector temperature was set at 300 °C. A 5 μL aliquot of the sample solution was injected, and quantification was performed using the internal standard method with hexadecyl hexadecanoate (3 mg/mL in n-hexane), as prescribed in GB 14756–2010. Injection volumes (1 μL for d-α-tocopheryl acetate and d-α-tocopheryl succinate; 5 μL for dl-α-tocopheryl acetate) were strictly applied as prescribed in each official method, since variations in injection volume directly affect peak area, calibration response, and consequently the contribution of injection variability to measurement uncertainty. The Japanese method employed HPLC–PDA analysis under the same chromatographic conditions used for d-α-tocopheryl acetate. A Phenomenex Hypersil C18 column (250 × 4.6 mm, 5 μm) maintained at 35 °C was used with an isocratic mobile phase of methanol:water (98:2, *v*/v). The flow rate was 1.0 mL/min, the injection volume was 20 μL, and detection was performed at 284 nm.

### 2.5 Analytical procedures for d-α-tocopheryl succinate

The methods reproduced for d-α-tocopheryl succinate follow the official procedures (primarily GC–FID in Korea and China) to allow direct method comparisons. GC–FID analysis was conducted using an HP-1 capillary column, with the injector temperature set at 290 °C and nitrogen (N₂) as the carrier gas. The oven temperature was maintained isothermally at 260 °C, and the detector temperature was set at 300 °C. A 1 μL aliquot of each sample solution was injected into the system. Prior to analysis, the sample was derivatized by reaction with methanol, 2,2-dimethoxypropane, and hydrochloric acid in the dark for 1 h, followed by evaporation under nitrogen, to convert the acid succinate to its methyl ester. The derivatized sample was then redissolved in the internal standard solution. Quantification was performed using the internal standard method with hexadecyl hexadecanoate (3 mg/mL in n-hexane) as the internal standard.

### 2.6 Method validation

The reproduced official methods for d-α-tocopheryl acetate, dl-α-tocopheryl acetate, and d-α-tocopheryl succinate were validated in accordance with ICH Q2(R1). Validation parameters included specificity, linearity, LOD, LOQ, precision, and accuracy. Specificity was evaluated by analyzing reagent blanks, matrix blanks, and standard-spiked samples. For chromatographic methods (HPLC and GC), no interfering peaks were observed at the analyte retention times. For the Korean titration method, matrix components did not affect the redox endpoint. Linearity was assessed using standard solution calibration curves prepared in the same solvent system as the sample at appropriate concentration ranges for each analyte and method. Linear regression was applied without weighting, as no significant heteroscedasticity was observed across the calibration range, and residual distribution was inspected to confirm the linearity assumption. Calibration curves showed excellent linearity with correlation coefficients exceeding 0.998 (R^2^ values are reported rounded to six decimal places; actual values were ≥ 0.999453 for all methods). LOD and LOQ were calculated using the equations LOD = 3.3(σ/S) and LOQ = 10(σ/S), based on the standard deviation of the response and the slope of the calibration curve. Specifically, σ corresponds to the standard deviation of the y-intercept of the calibration curve, as recommended by ICH Q2(R1), and S denotes the slope. Calibration curves were constructed using five concentration levels with three replicate injections per level for all three analytical methods (GC, HPLC, and titration), in accordance with ICH Q2(R1) guidance for linearity assessment. The reported LOD and LOQ values represent the method LOD/LOQ obtained from the complete analytical procedure (including sample preparation and the entire analytical workflow) rather than instrumental signal detection alone. For the titrimetric method, conventional σ/S-based LOD/LOQ are not directly applicable; instead, an equivalent sensitivity concept of minimum determinable content was considered, based on the burette resolution (0.05 mL) and the 0.01 N ammonium cerium(IV) sulfate titrant, corresponding to approximately 0.118 mg of dl-α-tocopheryl acetate per titration as the lower limit governed by endpoint uncertainty. Precision was assessed as repeatability and intermediate precision. Repeatability (*n* = 6) and inter-day precision (*n* = 3, over three days) were expressed as RSD%. All methods met the acceptance criteria of ≤5% for chromatographic methods and ≤ 10% for titration, in accordance with ICH Q2(R1) and AOAC International guidelines for analytical method validation at the relevant concentration ranges. Accuracy was determined through recovery experiments at low, medium, and high fortification levels. In addition, system suitability was assessed by evaluating the relative standard deviation (RSD) of the analytical signal (peak area for chromatographic methods, titrant volume for the titration method) obtained from triplicate replicate measurements of standard solutions at each calibration level, in accordance with ICH Q2(R1) recommendations. Although the present study did not include intentional robustness experiments (e.g., deliberate variation of flow rate, column temperature, or mobile-phase composition), this limitation is acknowledged in the Conclusion, since each method was reproduced strictly as prescribed by the respective official monograph. Overall, all official methods reproduced in this study satisfied the ICH Q2(R1) criteria for quantitative analysis. Replicate-level calibration data and validation results (intra-day and inter-day recovery and precision) for all reproduced methods are provided as Tables S1, S2, and S3 in the analyte-specific supplementary materials (S1: d-α-tocopheryl acetate; S2: dl-α-tocopheryl acetate; S3: d-α-tocopheryl succinate).

### 2.7 Measurement uncertainty

The measurement uncertainty of d-α-tocopheryl acetate, dl-α-tocopheryl acetate, d-α-tocopheryl succinate quantification was evaluated for each reproduced official method (GC–FID, HPLC–PDA, and titration). The estimation followed a metrological approach in accordance with the ISO/IEC Guide 98–3 (ISO, 1995), incorporating data from precision studies, analytical performance, and quantification results (NIST, 1993). The uncertainty sources include the reference material, analytical balance, volumetric apparatus, calibration curve, and instrument variation (Dias et al., 2011); for the titrimetric method, additional method-specific components such as titrant concentration standardization, endpoint detection, and volumetric burette resolution were also considered. The combined standard uncertainty was calculated by propagating the individual uncertainty components, and the expanded measurement uncertainty (U) was obtained by multiplying the combined standard uncertainty by a coverage factor (k = 2) corresponding to approximately 95% confidence level. The combined standard uncertainty (Eq. 1), the effective degrees of freedom estimated using the Welch–Satterthwaite formula (Eq. 2), and the expanded uncertainty (Eq. 3) were calculated using the following equations:

(1) $$u_{c}^{2}\left(y\right)=\sum_{i=1}^{N}{\left(\frac{\mathrm{\theta f}}{\theta x_{i}}\right)}^{2}u^{2}\left(x_{i}\right)$$

(2) $$V_{\mathrm{eff}}=\frac{u_{c}^{4}\left(y\right)}{\sum_{i=1}^{N}\frac{\left[c_{i}u{\left(x_{i}\right)}^{2}\right]}{v_{i}}}$$

(3) $$U\left(y\right)=ku_{c}\left(y\right)$$

*U*: Expanded uncertainty, k: Coverage factor, *V*_*eff*_: Effective degree of freedom, *v*_*i*_: Degree of freedom; u_*c*_(y): combined standard uncertainty of measurand y; ∂f/∂x_*i*_: sensitivity coefficient describing the influence of input quantity x_*i*_ on the output estimate; u(x_*i*_): standard uncertainty of input quantity x_*i*_; N: number of input quantities.

### 2.8 Statistical analysis

To quantitatively assess inter-method differences beyond conventional validation, a formal method-comparison framework was applied to the recovery (%) values obtained for dl-α-tocopheryl acetate, which was the only analyte determined by all three analytical principles (HPLC–PDA, GC–FID, and titration). One-way analysis of variance (ANOVA) was performed using the individual replicate recovery values (three concentration levels × three replicates, *n* = 9 per method), followed by Tukey's honestly significant difference (HSD) post-hoc test for pairwise comparisons. Statistical significance was defined as *p* < 0.05. Normality was confirmed using the Shapiro–Wilk test, and homogeneity of variances was evaluated using Levene's test. All statistical analyses were performed using Python (SciPy and statsmodels packages). Detailed group statistics, the ANOVA table, and Tukey HSD pairwise comparisons are provided in Table S2 (dl-α-tocopheryl acetate, ANOVA & Tukey sheet).

## 3 Results and discussion

### 3.1 Method validation for d-α-tocopheryl acetate

The reproduced GC and HPLC official methods for d-α-tocopheryl acetate were validated in accordance with ICH Q2(R1), and both approaches demonstrated adequate analytical performance. Since both Korea (MFDS) and China (GB) prescribe GC–FID for d-α-tocopheryl acetate under nearly identical operating conditions, GC results are presented as a unified GC–FID dataset, while the HPLC–PDA results correspond to the Japanese (MHLW) procedure. The calibration parameters, correlation coefficients, LOD, and LOQ for both methods are summarized in Table 1, and the corresponding precision (RSD) and accuracy (recovery) results at three concentration levels are presented in Table 2. Excellent linearity was obtained for the two methods, with the GC–FID calibration curve showing a correlation coefficient (R^2^) of 0.999453 over the concentration range of 750–12,000 μg/mL and the HPLC–PDA method exhibiting perfect linearity (R^2^ = 0.999992) across 125–2000 μg/mL. These results indicate that detector responses were proportional to analyte concentration within ranges relevant to regulatory compliance testing and quality control of tocopheryl acetate (Cho et al., 2007), which is typically present at relatively high levels in fortified foods and food additives. Comparable linear behavior for tocopheryl esters has been consistently reported in previous chromatographic studies, supporting the suitability of both GC and HPLC techniques for quantitative determination when appropriate calibration strategies are applied (Abidi, 2000; Rupérez et al., 2001). A pronounced difference was observed in analytical sensitivity between the two methods. The GC–FID method achieved markedly lower detection limits (LOD 0.02 μg/mL, LOQ 0.05 μg/mL) compared with the HPLC–PDA method (LOD 1.52 μg/mL, LOQ 4.61 μg/mL). This discrepancy can be attributed to differences in detection principles. Flame ionization detection provides a strong and nearly universal response for non-polar organic compounds (Abidi, 2000), making it particularly sensitive for lipid-soluble analytes such as d-α-tocopheryl acetate. In contrast, PDA detection relies on chromophoric absorption, which is relatively weak for tocopheryl esters, resulting in higher detection limits (Liu et al., 2025). Similar sensitivity trends between GC–FID and HPLC–PDA have been reported for vitamin E derivatives and other fat-soluble vitamins, confirming that GC–FID generally offers superior sensitivity for such compounds (Arias-Santé et al., 2024; Huo et al., 1999). Precision evaluation further supported the reliability of both analytical procedures (Niero et al., 2018). For the GC method, intraday relative standard deviation (RSD) values ranged from 0.34% to 1.31%, while interday RSD values ranged from 1.01% to 2.68%, indicating satisfactory repeatability and intermediate precision. The HPLC method demonstrated even tighter precision, with intraday RSD values between 0.66% and 1.61% and exceptionally low interday RSD values (0.21–0.79%). These results suggest that both methods are capable of producing reproducible quantitative data, although the liquid-phase separation employed in HPLC provides enhanced stability under routine analytical conditions. Accuracy assessment revealed characteristic method-dependent differences. The GC method exhibited a relatively wide recovery range (86.34–114.86%), particularly at higher concentration levels where recoveries tended to exceed 110%. This behavior suggests that quantitative accuracy in GC analysis may be influenced by injection-to-injection variability and the thermal behavior of d-α-tocopheryl acetate, including potential volatilization or adsorption effects under elevated injector and oven temperatures. Similar recovery variability has been reported in previous GC-based analyses of tocopherol esters, where high analytical sensitivity was accompanied by increased susceptibility to concentration-dependent bias (Good et al., 2005). In contrast, the HPLC–PDA method showed highly stable recoveries ranging from 96.67% to 99.75% across all concentration levels, consistently meeting typical acceptance criteria for quantitative chromatographic analysis. The mild thermal conditions and liquid-phase separation employed in HPLC likely contribute to the observed robustness in quantitative performance. Overall, both GC–FID and HPLC–PDA methods satisfied the essential validation requirements for the quantification of d-α-tocopheryl acetate. However, the two methods exhibited distinct analytical characteristics: GC–FID provided superior sensitivity, whereas HPLC–PDA offered more consistent accuracy and robustness across concentration levels. These findings demonstrate that differences in analytical performance are intrinsically linked to the underlying detection principles and methodological design. Consequently, such method-dependent characteristics should be carefully considered when comparing quantitative results obtained using different national official procedures or when selecting analytical methods for routine determination of d-α-tocopheryl acetate (Kong et al., 2011). Notably, the official GC–FID methods of both Korea (MFDS) and China (GB) prescribe the use of hexadecyl hexadecanoate (cetyl palmitate; CAS 540–10-3, at 3 mg/mL in n-hexane) as an internal standard to compensate for injection variability. The Japanese (MHLW) HPLC–PDA methods, in contrast, rely on external calibration since liquid-phase separation provides inherently lower injection-related variability. Despite the use of internal standardization in the present GC–FID analyses, the recovery range was still relatively wide (86.34–114.86%), indicating that additional sources of variability beyond injection reproducibility-such as the thermal behavior of d-α-tocopheryl acetate under elevated injector/oven temperatures and split-injection effects-contribute substantially to GC–FID quantitative performance. These observations are consistent with the GC–FID-dominant uncertainty contribution attributed to calibration and injection in the uncertainty budget (Section 3.4). More broadly, validated analytical techniques are essential for ensuring reproducibility and accuracy in food quality assessment, particularly when method-dependent biases may influence quantitative outcomes (Akram et al., 2024).

**Table 1:** Correlation coefficients of the calibration curves, and limit of detection (LOD) and limit of quantification (LOQ) of d-α-tocopheryl acetate analysis.

Analyte	Method	Range (μg/mL)	Slope	Intercept	Correlation coefficient (R^2^)	LOD (μg/mL)	LOQ (μg/mL)
d-α-tocopheryl acetate	GC	750–12,000	0.0003	−0.0026	0.999453	0.02	0.05
	HPLC	125–2000	3911.6543	31,118.014	0.999992	1.52	4.61

**Table 2:** Precision (RSD) and accuracy (Recovery) of d-α-tocopheryl acetate analysis.

Analyte			Concentration (μg/mL)	Mean ± SD (μg/mL)	RSD (%)	Recovery (%)
d-α-tocopheryl acetate	GC	Intra-day	1500	1487.38 ± 8.28	0.56	93.89
			3000	3132.31 ± 41.10	1.31	86.34
			6000	6143.56 ± 20.87	0.34	114.51
		Inter-day	1500	1543.06 ± 21.94	1.42	94.22
			3000	3045.07 ± 81.55	2.68	86.55
			6000	5964.23 ± 60.30	1.01	114.86
	HPLC	Intra-day	250	242.52 ± 3.92	1.61	97.01
			500	496.33 ± 3.31	0.66	99.27
			1000	997.51 ± 6.72	0.67	99.75
		Inter-day	250	241.68 ± 0.09	0.21	96.67
			500	495.90 ± 0.20	1.01	99.18
			1000	996.90 ± 0.08	0.79	99.70

### 3.2 Method validation for dl-α-tocopheryl acetate

The GC, HPLC, and titration methods reproduced for the determination of dl-α-tocopheryl acetate were validated in accordance with ICH Q2(R1), and all three approaches demonstrated adequate analytical performance. Table 3 summarizes the calibration parameters, correlation coefficients, LOD, and LOQ obtained for the GC, HPLC, and titration methods, while Table 4 presents the precision (RSD) and accuracy (recovery) results at three concentration levels. Excellent linearity was obtained across the designated calibration ranges for each method. The GC–FID method exhibited a correlation coefficient (R^2^) of 0.999967 over the concentration range of 750–12,000 μg/mL, while the HPLC–PDA method showed perfect linearity (R^2^ = 0.999949) between 125 and 2000 μg/mL. The titration method also demonstrated ideal linearity (R^2^ = 0.999999) across 312.5–5000 μg/mL, confirming a proportional relationship between titrant consumption and analyte concentration. These results indicate that dl-α-tocopheryl acetate, which is typically supplied and regulated as a racemic ester at relatively high concentrations (JRC, 2010), can be reliably quantified by all three analytical principles within ranges relevant to regulatory testing and routine quality control (Castan et al., 2005). Analytical sensitivity differed depending on the technique employed. The GC–FID method yielded LOD and LOQ values of 1.50 and 4.53 μg/mL, respectively, whereas the HPLC–PDA method exhibited superior sensitivity with lower LOD and LOQ values of 0.65 and 1.96 μg/mL. Although flame ionization detection generally provides a strong response for non-polar organic compounds, the relatively lower sensitivity observed for GC in this case may be attributed to the broader chromatographic peaks and injection-related variability associated with the analysis of racemic dl-α-tocopheryl acetate (Pasias et al., 2018). In contrast, liquid-phase separation combined with PDA detection appears to provide enhanced signal stability for this compound, resulting in lower detection limits. As expected for a wet-chemical redox assay, the titration method does not yield instrumentally derived LOD or LOQ values, as sensitivity is governed by reaction stoichiometry and endpoint detection rather than signal-to-noise considerations. Precision evaluation demonstrated that all three methods provided satisfactory repeatability and intermediate precision. The GC method showed intraday RSD values ranging from 0.34% to 1.56% and interday RSD values between 1.56% and 2.18%, indicating acceptable reproducibility for chromatographic determination at high analyte concentrations. The HPLC method exhibited the tightest precision, with intraday RSD values of 0.70–1.61% and exceptionally low interday RSD values of 0.01–0.08%, reflecting the stability of liquid-phase separation and PDA detection for dl-α-tocopheryl acetate (Niero et al., 2018). The titration method also demonstrated stable precision, with intraday RSD values of 0.30–0.60% and interday RSD values of 0.45–1.54%, consistent with the robustness of classical redox titration when applied to high-concentration analytes. Accuracy assessment further revealed method-dependent characteristics. GC recoveries ranged from 100.76% to 106.01%, suggesting a slight positive bias that may arise from injection variability or thermal behavior of dl-α-tocopheryl acetate under GC conditions. HPLC recoveries were slightly lower but more consistent, ranging from 94.13% to 97.02%, indicating stable quantitative performance with minimal concentration dependent bias. Notably, the titration method produced highly consistent recoveries close to 100% across all tested concentrations (99.86–100.45%), reflecting the stoichiometric nature of the redox reaction and the suitability of titrimetric analysis for accurate determination of dl-α-tocopheryl acetate at moderate to high concentration levels. Overall, all three national official methods demonstrated strong analytical capability for the quantification of dl-α-tocopheryl acetate. However, distinct method-dependent characteristics were evident. The GC method yielded high recoveries but slightly broader variability, the HPLC method provided the highest precision and sensitivity, and the titration method showed robust and accurate performance suitable for routine quantification where dl-α-tocopheryl acetate is present at relatively high concentrations. These findings demonstrate that differences in analytical performance are primarily governed by methodological design and detection principles rather than validation adequacy (Apak et al., 2016; Huang et al., 2025), and such characteristics should be carefully considered when comparing national analytical procedures for dl-α-tocopheryl acetate. To formally quantify these inter-method differences, a one-way ANOVA was performed on the individual replicate recovery values (three concentration levels × three replicates, *n* = 9 per method). The mean recoveries differed significantly among the three methods (F = 40.64, *p* < 0.001), with mean values of 96.03 ± 1.17% (HPLC–PDA), 103.56 ± 2.80% (GC–FID), and 100.18 ± 0.47% (titration). Tukey's HSD post-hoc test confirmed that all three pairwise comparisons were statistically significant (GC vs. HPLC: mean difference + 7.52%, p < 0.001; GC vs. titration: +3.37%, *p* = 0.001; HPLC vs. titration: −4.15%, p < 0.001). These results indicate that GC–FID exhibited a positive bias whereas HPLC–PDA showed a slight negative bias relative to titration, which produced recovery values closest to the nominal 100%. Notably, despite the statistically significant differences, all recovery values fell within the conventional ICH Q2(R1) acceptance range (typically ±10%), supporting practical equivalence for routine quality-control applications while demonstrating that structural method-dependent biases persist even when each method individually meets validation criteria. Comparable HPLC-based validation frameworks—incorporating linearity, LOD/LOQ, and precision evaluation—have been recently applied to the quantification of polar food constituents in common fruits and vegetables, further supporting the robustness of HPLC for routine food-component analysis (Lim et al., 2025).

**Table 3:** Correlation coefficients of the calibration curves, and limit of detection (LOD) and limit of quantification (LOQ) of dl-α-tocopheryl acetate analysis.

Analyte	Method	Range (μg/mL)	Slope	Intercept	Correlation Coefficient (R^2^)	LOD (μg/mL)	LOQ (μg/mL)
dl-α-tocopheryl acetate	GC	750–12,000	0.0002	0.0124	0.999967	1.50	4.53
	HPLC	125–2000	4421.1	56,329.0	0.999949	0.65	1.96
	Titration	312.5–5000	0.0204	−0.2222	0.999999	–	–

**Table 4:** Precision (RSD) and accuracy (Recovery) of dl-α-tocopheryl acetate analysis.

Analyte			Concentration (μg/mL)	Mean ± SD (μg/mL)	RSD (%)	Recovery (%)
dl-α-tocopheryl acetate	GC	Intra-day	1500	1532.86 ± 31.56	1.56	102.20
			3000	3180.15 ± 51.10	1.31	106.01
			6000	6280.52 ± 90.87	0.34	104.51
		Inter-day	1500	1511.40 ± 32.99	2.18	100.76
			3000	3118.70 ± 53.34	1.71	103.96
			6000	6357.22 ± 99.74	1.56	105.95
	HPLC	Intra-day	250	241.42 ± 0.55	1.61	96.57
			500	470.67 ± 0.55	0.66	94.13
			1000	964.70 ± 6.72	0.70	94.47
		Inter-day	250	242.55 ± 0.05	0.02	97.02
			500	472.45 ± 0.37	0.08	94.49
			1000	965.91 ± 3.79	0.01	96.60
	Titration	Intra-day	625	625.39 ± 4.89	0.45	100.06
			1250	1248.37 ± 7.47	0.60	99.87
			2500	2497.60 ± 7.45	0.30	99.90
		Inter-day	625	627.82 ± 2.83	0.45	100.45
			1250	1252.91 ± 27.50	1.54	100.23
			2500	2496.55 ± 4.91	0.83	99.86

### 3.3 Method validation for d-α-tocopheryl succinate

The GC method reproduced for the determination of d-α-tocopheryl succinate was validated in accordance with ICH Q2(R1) and demonstrated adequate analytical performance. Table 5 summarizes the calibration parameters, correlation coefficient, LOD, and LOQ obtained for the GC method, and Table 6 presents the corresponding precision (RSD) and accuracy (recovery) data at three concentration levels. Excellent linearity was obtained over the concentration range of 750–12,000 μg/mL, with a correlation coefficient (R^2^) of 0.999691, indicating a proportional detector response across concentration levels relevant to regulatory testing and quality control (Bowman & West, 1968; Mahn et al., 1968). Compared with tocopheryl acetate derivatives, d-α-tocopheryl succinate possesses a bulkier ester moiety and increased polarity, yet the observed linearity confirms that GC–FID can reliably quantify this compound when appropriate chromatographic conditions are applied(Rupérez et al., 2001). The method exhibited high analytical sensitivity, yielding an LOD of 0.01 μg/mL and an LOQ of 0.036 μg/mL. This high sensitivity reflects the strong response of flame ionization detection toward esterified tocopherol derivatives containing multiple hydrocarbon moieties (Rupérez et al., 2001). Similar sensitivity has been reported in previous GC-based studies on tocopheryl succinate and related ester derivatives, supporting the suitability of GC–FID for trace-level determination of this compound (Good et al., 2005). These results indicate that, despite the increased molecular complexity of the succinate ester, efficient ionization in the flame allows sensitive detection under optimized GC conditions. Precision evaluation showed acceptable repeatability and intermediate precision. Intra-day RSD values ranged from 0.24% to 2.59%, while inter-day RSD values ranged from 0.61% to 1.54%. The relatively higher RSD values observed at the lowest concentration level (1500 μg/mL) may be attributed to increased susceptibility to injection variability and subtle fluctuations in chromatographic behavior at lower signal intensities. Nevertheless, all RSD values remained within generally accepted limits for quantitative GC analysis, indicating that the method provides reproducible results across the tested concentration range. Accuracy assessment revealed recovery values ranging from 85.61% to 96.69% for intraday measurements and from 86.21% to 91.97% for inter-day measurements. These results indicate a tendency toward slight underestimation at certain concentration levels. Such behavior may be associated with the thermal stability and volatilization characteristics of d-α-tocopheryl succinate (Souza et al., 2022), as the succinate ester group can influence analyte transfer efficiency during injection and passage through the GC system. Compared with tocopheryl acetate, succinate esters are known to exhibit stronger intermolecular interactions and reduced volatility, which can contribute to incomplete vaporization or adsorption-related losses under GC conditions. Similar under-recovery trends have been reported in previous studies examining the chromatographic behavior of tocopheryl succinate derivatives (Good et al., 2005). Overall, the reproduced GC method demonstrated strong linearity, high sensitivity, and acceptable precision and accuracy for the quantification of d-α-tocopheryl succinate. However, the observed variability and slight negative bias at lower concentrations highlight the influence of analyte-specific physicochemical properties on GC-based quantification. These findings indicate that, while GC–FID is suitable for the determination of d-α-tocopheryl succinate, method performance is inherently affected by the structural characteristics of the succinate ester (Gamna & Spriano, 2021). Consequently, such analyte-dependent factors should be carefully considered when interpreting quantitative results or comparing national analytical procedures for tocopheryl succinate.

**Table 5:** Correlation coefficients of the calibration curves, and limit of detection (LOD) and limit of quantification (LOQ) of d-α-tocopheryl succinate analysis by GC.

Analyte	Range (μg/mL)	Slope	Intercept	Correlation Coefficient (R^2^)	LOD (μg/mL)	LOQ (μg/mL)
d-α-tocopheryl succinate	750–12,000	0.0001	−0.0366	0.999691	0.01	0.036

**Table 6:** Precision (RSD) and accuracy (Recovery) of d-α-tocopheryl succinate analysis by GC.

Analyte		Concentration (μg/mL)	Mean ± SD (μg/mL)	RSD (%)	Recovery (%)
d-α-tocopheryl succinate	Intra-day	1500	1319.13 ± 1.76	2.59	87.94
		3000	2568.31 ± 0.73	0.24	85.61
		6000	5801.34 ± 1.44	0.35	96.69
	Inter-day	1500	1379.59 ± 0.62	0.61	91.97
		3000	2642.52 ± 1.33	1.54	88.08
		6000	5172.46 ± 1.30	0.83	86.21

### 3.4 Measurement uncertainty

**Fig. 1:**
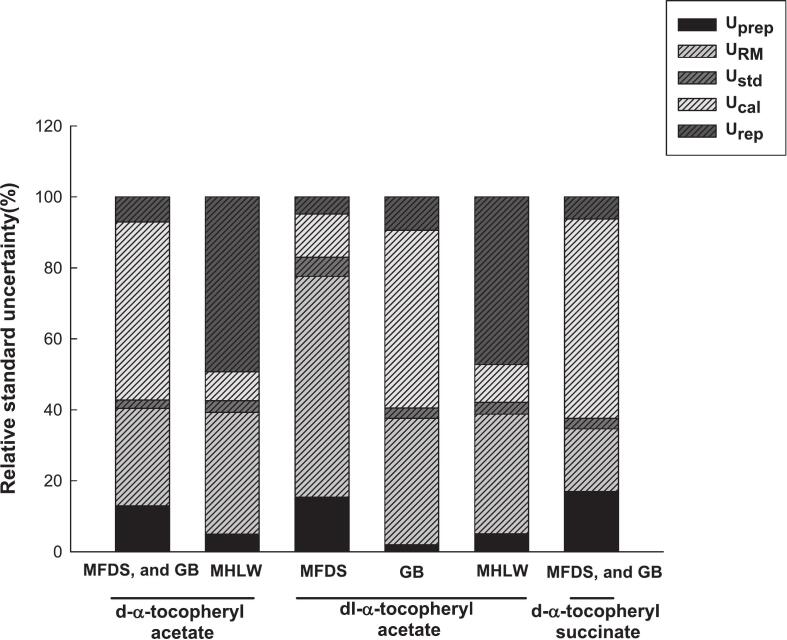
**Contribution of each source of uncertainty for tocopheryl esters analysis.** U_prep_, sample preparation; U_RM_, reference material; U_std_, standard stock solution; U_cal_, calibration curve; and U_rep_, repeatability. Uncertainty profiles were additionally evaluated for d-α-tocopheryl acetate, dl-α-tocopheryl acetate, and d-α-tocopheryl succinate, with all components normalized to 100% for cross-method comparison.

The relative contributions of individual uncertainty sources for the determination of tocopheryl esters using national official methods are summarized in Fig. 1 and Table 7 (the complete uncertainty budget with all component values and calculation formulas is provided in the Uncertainty sheet of each analyte-specific supplementary file: Table S1 for d-α-tocopheryl acetate, Table S2 for dl-α-tocopheryl acetate, and Table S3 for d-α-tocopheryl succinate). Although all measurements were conducted using identical fortified samples under controlled laboratory conditions, the uncertainty profiles differed substantially depending on the analytical procedure applied. This finding demonstrates that variability among national methods is not merely a consequence of random analytical error but is intrinsically linked to the structural design of each official procedure. According to the ISO GUM framework, the combined standard uncertainty reflects the propagation of uncertainty arising from key analytical steps, including calibration, sample preparation, and repeatability. Therefore, the dominance of a specific uncertainty component can be interpreted as indicating which step most strongly governs result comparability when the method is applied in a regulatory context (Štěpán et al., 2004). In this regard, the present results provide insight into why quantitative discrepancies may persist even when official methods individually satisfy conventional validation criteria (Djellouli et al., 2018). For d-α-tocopheryl acetate, clear differences were observed between chromatographic methods. The GC–FID based procedure exhibited a pronounced contribution from calibration-related uncertainty, reflecting the sensitivity of detector response to injection reproducibility and calibration slope stability (Komsta et al., 2021). This behavior is consistent with the inherent characteristics of FID-based quantification, where small variations in injected mass can directly influence signal intensity and regression parameters. In contrast, the HPLC-based method showed a higher contribution from sample preparation–related uncertainty, suggesting that volumetric operations and solution handling exert a stronger influence on the overall reliability of the quantified result than instrumental signal variability. These observations indicate that, even for the same analyte, the dominant source of uncertainty shifts depending on the analytical principle employed. A similar method-dependent pattern was observed for dl-α-tocopheryl acetate. The titrimetric procedure demonstrated a comparatively higher contribution from repeatability-related uncertainty, which can be attributed to the manual and reaction-based nature of redox titration, including endpoint recognition and operator-dependent variability. In contrast, instrumental methods relied more heavily on calibration and preparation components, indicating that quantitative performance was governed primarily by procedural design rather than random instrumental noise. This distinction highlights that analytical uncertainty is shaped not only by instrument capability but also by the fundamental measurement concept underlying each method. For d-α-tocopheryl succinate, uncertainty contributions were predominantly associated with sample preparation and calibration across the evaluated methods. This tendency may be related to the physicochemical properties of the succinate ester, such as increased polarity and altered handling behavior, which can influence extraction efficiency and chromatographic response stability. Consequently, method-dependent uncertainty structures became more pronounced for compounds with greater structural complexity, underscoring the importance of analyte-specific considerations when evaluating official procedures. Taken together, these results demonstrate that national official analytical methods differ not only in detection principles but also in the distribution and origin of analytical uncertainty. Importantly, this study shows that compliance with standard validation parameters alone does not guarantee equivalence in quantitative reliability across methods. Measurement uncertainty was therefore applied not as a primary objective but as an interpretative framework to elucidate the structural reasons underlying method-dependent variability (De Beer et al., 2003; Dias et al., 2011). From a regulatory and food analysis perspective, these findings emphasize that comparative evaluation of official methods should consider uncertainty structure in addition to validation performance, particularly when analytical results are used for cross-national comparison, regulatory decision-making, or harmonization of food additive standards (Separovic and Lourenço, 2018). The importance of analytical accuracy and uncertainty awareness extends beyond food additive testing to broader chemical safety assessment, where measurement reliability directly governs the interpretation of contaminant exposure and bioaccumulation data in biological systems (Gill et al., 2025). Consistent with the present analysis, a recent ISO-GUM-based evaluation of an HPLC-UV assay also identified calibration and sample volume as the dominant uncertainty contributors, underscoring that uncertainty structure—rather than validation alone—governs analytical reliability across regulatory contexts (Haidara et al., 2025). These findings suggest that harmonization efforts should consider not only analytical performance criteria but also the dominant sources of uncertainty embedded in official methods. From a practical standpoint, the present findings have direct implications for routine food additive quality control and regulatory monitoring: laboratories operating under different national frameworks may report systematically different quantitative outcomes for the same product, and uncertainty-aware reporting can therefore enhance cross-laboratory comparability and support more reliable compliance decisions. Furthermore, the integration of measurement-uncertainty interpretation with conventional validation provides a more comprehensive framework for evaluating analytical reliability, which is increasingly emphasized in fortified food and nutraceutical analysis where quantitative accuracy underpins product safety and labeling compliance. Such analytical reliability is equally relevant for the quality assessment of functional beverages and fortified products, where accurate quantification of bioactive constituents directly influences nutritional labelling and storage-stability evaluation (Ishaq et al., 2025). Similarly, validated HPLC procedures employing certified reference materials for verifying recovery have been recently applied to vitamin determination in food products, supporting the construction of national food-composition databases (Je et al., 2026). Such validated and uncertainty-aware analytical approaches are increasingly important across food chemistry, where sensitive and reliable quantification underpins food safety, regulatory compliance, and accurate determination of bioactive and additive compounds (Nadeem et al., 2025).

**Table 7:** Individual uncertainties of the sample preparation (U_prep_), reference material (U_RM_), standard stock solution (U_std_), calibration curve (U_cal_), repeatability (U_rep_), and expanded uncertainty (U) according to the Eurachem Guide.

Analytes	U_prep_	U_RM_	U_std_	U_cal_	U_rep_	U
d-α-tocopheryl acetate (MFDS, GB)	0.0114	0.0241	0.0020	0.0441	0.0062	0.1039
d-α-tocopheryl acetate (MHLW)	0.0035	0.0241	0.0023	0.0057	0.0346	0.0855
dl-α-tocopheryl acetate (MFDS)	0.0057	0.0231	0.0020	0.0045	0.0018	0.0487
dl-α-tocopheryl acetate (GB)	0.0013	0.0231	0.0019	0.0325	0.0061	0.0808
dl-α-tocopheryl acetate (MHLW)	0.0035	0.0231	0.0023	0.0073	0.0324	0.0813
d-α-tocopheryl succinate (MFDS, GB)	0.0114	0.0119	0.0020	0.0378	0.0042	0.0830

## 4 Conclusion

This study reproduced the official analytical methods established in Korea, China, and Japan for the determination of three tocopheryl esters d-α-tocopheryl acetate, dl-α-tocopheryl acetate, and d-α-tocopheryl succinate and evaluated their analytical performance under standardized laboratory conditions. All reproduced methods satisfied the ICH Q2(R1) criteria for specificity, linearity, sensitivity, precision, and accuracy, demonstrating the fundamental reliability of each national procedure despite differences in analytical principles, instrumentation, and sample preparation. For d-α-tocopheryl acetate, the MFDS and GB GC–FID methods (Korea and China) produced consistent quantitative results with high reproducibility, while the MHLW HPLC–PDA method (Japan) exhibited slightly different sensitivity and recovery profiles attributable to differences in detection principles. The determination of dl-α-tocopheryl acetate showed the expected variability in the titration-based Korean method, whereas the chromatographic methods used in China and Japan provided more consistent results. d-α-Tocopheryl succinate, analyzed by GC in both Korea and China, showed overall agreement between the two methods with only minor deviations. Collectively, these findings indicate that official analytical methods used across different countries can yield generally consistent outcomes, yet measurable differences may arise depending on methodological structure and analytical approach. Such variability highlights the importance of continuous review, harmonization, and scientific reassessment of official food additive test methods across regulatory authorities. The comparative evaluation performed in this study provides meaningful foundational data that may support future development, refinement, and harmonization of analytical standards for tocopheryl esters in food additive regulations. In particular, statistical analysis (one-way ANOVA, F = 40.64, *p* < 0.001) confirmed that recovery values for dl-α-tocopheryl acetate differed significantly among the three analytical methods, with all values nevertheless remaining within the conventional ICH Q2(R1) acceptance range, indicating practical equivalence for routine quality-control use despite structural method-dependent biases. Several limitations of the present study should be acknowledged. First, the analyzed samples were limited to food-grade tocopheryl ester additive products, and the methods were not evaluated against real food matrices such as vegetable oils or vitamin-fortified foods, which may introduce additional variability through matrix effects and extraction efficiency. Second, although measurement uncertainty was estimated using ISO GUM and Eurachem approaches, broader inter-laboratory reproducibility was not directly examined. Third, formal robustness testing (e.g., deliberate variation of flow rate, column temperature, or wavelength) was not performed, as each method was reproduced strictly as prescribed by the respective official monograph; system suitability, however, was confirmed for all methods through replicate-injection RSD evaluation. Future studies extending these comparisons to real fortified food matrices, integrating advanced analytical technologies such as automated chromatography, mass spectrometry-based platforms, and data-driven analytical approaches, would further strengthen the reliability and harmonization of analytical methods used in food additive testing and food composition databases. In parallel, the broader landscape of modern food analytical science increasingly integrates microbial and biotechnological monitoring with chromatographic and spectroscopic platforms (Wijaya et al., 2026), suggesting that future cross-disciplinary integration will play a key role in advancing analytical capability for food additive and composition analysis.

## CRediT authorship contribution statement

**Geun-Hee Cho:** Writing – original draft, Validation, Methodology, Conceptualization. **Hyun-Woo Oh:** Validation, Data curation. **Tae-Woong Song:** Resources, Formal analysis. **Young-Jae Heo:** Validation, Formal analysis. **Su-Jong Kim:** Formal analysis. **Ji-Hyun Im:** Validation, Formal analysis. **Xiaolu Fu:** Visualization, Validation. **June-Seok Lim:** Visualization. **Min-Hye Kim:** Validation. **Hee-Jae Suh:** Writing – review & editing, Funding acquisition. **Ok-Hwan Lee:** Writing – review & editing, Funding acquisition. **Sun-Il Choi:** Writing – review & editing, Conceptualization.

## Declaration of competing interest

The authors declare that they have no known competing financial interests or personal relationships that could have appeared to influence the work reported in this paper.

## Data Availability

Data will be made available on request.

## Appendix A Supplementary data

Supplementary material Replicate-level calibration, recovery, and precision data for d-α-tocopheryl acetate (Table S1), dl-α-tocopheryl acetate (Table S2), and d-α-tocopheryl succinate (Table S3), obtained using the reproduced Korean (MFDS), Chinese (GB), and Japanese (MHLW) official methods (GC–FID, HPLC, and titration).
